# Life history of a brain autoreactive T cell: From thymus through intestine to blood-brain barrier and brain lesion

**DOI:** 10.1016/j.neurot.2024.e00442

**Published:** 2024-09-04

**Authors:** Naoto Kawakami, Hartmut Wekerle

**Affiliations:** a Institute of Clinical Neuroimmunology, University Hospital, LMU Munich and Biomedical Center (BMC), Faculty of Medicine, LMU Munich, Germany; b Emeritus Group Neuroimmunology, Max Planck Institute of Biological Intelligence, Germany

**Keywords:** Central nervous system, Autoimmunity, CNS-specific T cells, Experimental autoimmune encephalomyelitis, Microbiota

## Abstract

Brain antigen-specific autoreactive T cells seem to play a key role in inducing inflammation in the central nervous system (CNS), a characteristic feature of human multiple sclerosis (MS). These T cells are generated within the thymus, where they escape negative selection and become integrated into the peripheral immune repertoire of immune cells. Typically, these autoreactive T cells rest in the periphery without attacking the CNS. When autoimmune T cells enter gut-associated lymphatic tissue (GALT), they may be stimulated by the microbiota and its metabolites. After activation, the cells migrate into the CNS through the blood‒brain barrier, become reactivated upon interacting with local antigen-presenting cells, and induce inflammatory lesions within the brain parenchyma. This review describes how microbiota influence autoreactive T cells during their life, starting in the thymus, migrating through the periphery and inducing inflammation in their target organ, the CNS.

## Introduction

Multiple sclerosis (MS) is commonly considered an autoimmune disease in which central nervous system (CNS)-autoreactive T cells drive an inflammatory response against cerebral white and gray matter [[1], [2], [3]]. This concept is based upon clinical and neuropathological observations supported by experimental animal studies, such as studies of experimental autoimmune encephalomyelitis (EAE). These studies have identified brain antigen-specific CD4^+^ T cells as crucial agents for inducing CNS inflammation [[4], [5], [6]]. This inflammation is followed by lesion formation amplified by other immune cells, such as cytotoxic T cells, autoantibody-producing B cells, and activated macrophages as well as brain resident microglia [7]. These immune cells were previously thought to cooperate autonomously. However, recent studies have shown that the function and fate of immune cells are influenced remotely by the gut microbiota. Therefore, the microbiota controls the pathogenesis of MS/EAE [8].

The gut-associated lymphatic tissue (GALT), the site where CD4^+^ T cells are physically located near the intestinal microbiota, harbors the body's largest assembly of immune cells [9]. Traditionally, this enormous region of immune reactivity was related to the intestine's dedicated function: the uptake and metabolism of nutrients. These compounds are processed both by secreted products of the body itself, i.e., from gut walls and surrounding exocrine tissues, and by the microbial community within the intestinal lumen. In addition, the GALT is required to ward off the omnipresent infectious microbes that besiege the body. Furthermore, diets and microbes represent foreign antigenic structures that need to be inspected by immune cells to tolerate benevolent structures (nutrients and microbial gut flora) but to exclude dangerous agents. More recent observations greatly extend this concept. It has become clear that the GALT profoundly shapes the immune system both functionally and morphologically [10]. Intestinal immune responses determine the body's immune tolerance or, in contrast, spark autoimmune diseases.

This review will retrace the autoimmune mechanisms that ultimately result in human MS, especially focusing on the life of brain-specific T cells, beginning with their education in the thymus and following the manifold triggers in the periphery that guide autoimmune T cells into the CNS, where they unleash the development of pathogenic lesions. We shall pay particular attention to the multiple steps that are under the influence of the gut.

## Autoimmune responses in the CNS

MS is considered an autoimmune response, although the specific autoantigen involved has not yet been identified. In the steady state, there are very few immune cells in the CNS. In addition, the CNS is thought to lack classic lymphatic drainage, and the blood‒brain barrier (BBB) separates the CNS from the peripheral circulation. Therefore, the CNS is considered an immune-privileged organ. The concept of immune privilege in the CNS has been challenged in recent years [11]. Louveau et al. demonstrated that lymphatic vessels from the CNS to the periphery transport cells and substances [12]. The BBB becomes permeable during inflammation, and particular immune cells can cross it, as we have previously shown [13]. Indeed, massive immune cell infiltration and perivascular cuffs have been observed in the CNS of MS patients. These findings suggest that the CNS is partly immune-privileged but not completely immune-free. Therefore, under special conditions, immune cells can infiltrate the CNS and induce local inflammation.

Once CD4^+^ T cells enter the CNS and recognize their target autoantigen, they produce inflammatory cytokines, which increase BBB permeability and recruit other immune cells to inflammatory lesions [14]. The role of CD4^+^ T cells, as initiators of human MS, is supported by several lines of evidence. In the seminal study by Madsen et al. [15], transgenic mice expressing T cell receptor (TCR) from an MS patient, HLA-DR2 as an MS susceptibility gene, and human CD4 were used, and the results showed that some of the mice developed spontaneous EAE. More directly, adoptive transfer of CNS antigen specific T cells induce the inflammation in the CNS [4,6,16], which present key features shared with early active MS lesions. In addition, an early genome-wide study identified IL2RA, IL17A and HLAs as major risk factors for MS [17] and a later genome-wide study of MS patients identified risk loci significantly associated with immune cells [18].

## Autoantigens in humans and rodents

Classically, autoimmune diseases are defined by the presence of plasma immunoglobulins (autoantibodies) that bind antigenic structures within the failing target organ. Identification of pathogenic autoantigens has mostly relied on screening of autoreactive immunoglobulin circulating in the bloodstream, where typically one set of autoantibodies recognizes one special target autoantigen. Classical examples of neurological autoimmune diseases include myasthenia gravis [19] and autoimmune encephalitis [20], which are caused by antibodies binding to defined determinants of synaptic proteins, thereby interfering with electrical signal transmission. In MS, antibodies, which form pathognomonic oligoclonal bands (OCBs) in the CSF of MS patients, are synthesized locally by B cells within the CSF [21]. A subsequent study reconstituted 6 antibodies from OCBs and identified three target antigens, which, however, were ubiquitously expressed intracellular proteins but not myelin antigens [22]. Some autoantibodies that were identified in patients with neurological symptoms were further examined in detail. For example, neuromyelitis optica spectrum disorder (NMOSD) was once considered a subtype of MS but is now recognized as a separate disease since NMOSD patients have anti-aquaporin 4 (AQP4) antibodies [23,24]. The AQP4 antibody serves not only as a biomarker for NMOSD but also as a pathogenic antibody, as shown in an animal model [25,26]. A number of potential autoantigens have been reported by various groups, but these have been restricted to minor subgroups of MS patients [27].

What are the target antigens of autoimmune T cells? Similar to type-1 diabetes mellitus [28] and rheumatoid arthritis [29], MS is considered a T cell-mediated disease [30,31]. This motivated numerous investigators to identify the target autoantigens recognized by pathogenic immune cells. Bielekova et al. demonstrated the existence of CD4^+^ T cells, which have high avidity against peptides from major myelin proteins, and further showed that the numbers of these T cells were greater in MS patients than in controls [32]. These myelin-specific T cells produce inflammatory cytokines, such as IFNγ, IL-17, and GM-CSF, after stimulation [33,34]. Inflammatory cytokines contribute to lesion formation and may also support autoantibody production by B cells. However, pathognomonic MS autoantigens still remain to be identified.

T and B cells work together to induce inflammatory lesions. For example, an anti-MOG antibody, which was isolated from a patient with myelin oligodendrocyte glycoprotein antibody-associated disease (MOGAD), was administered to recipient animals after adoptive transfer of MOG-specific T cells. This resulted in increased T cell infiltration and clear demyelination in the CNS [35]. According to Flach et al., an anti-MOG antibody targeted MOG from the CSF on local APCs and presented to anti-MOG-specific T cells, which resulted in the formation of larger lesions [36]. A compatible picture was noted in CNS lesions of people after therapeutic immunization with lyophilized calf brain tissue showed induced demyelination [37]. Neuronal antigen-specific T cells have also been implicated in peripheral nerve disorders. Recently, Th1 autoreactive T cells have been shown to act in a subset of patients with Guillain–Barré syndrome (GBS) [38]. These T cells may work together with autoantibodies like in anti-MOG autoimmunity.

Although the autoantigenic target in MS has remained elusive, a set of experimental animal EAE models have provided the first formal evidence of CNS-directed T cell-mediated autoimmune disease and have become an indispensable model for studying the immune mechanism of MS induction [39,40]. EAE was first produced in monkeys by repeated immunization with rabbit brain homogenate. The lesions were characterized by perivascular infiltrates and demyelination, reminiscent of active MS plaques [41]. Myelin basic protein (MBP) was identified as the critical pathogen-inducing autoantigen [42]. Formal evidence has revealed the isolation and generation of T cell lines from immunized [43] and even naïve [44] inbred rats and their potential to transfer EAE to recipients of the same strain. MBP-specific T cells were later identified in human peripheral blood [[45], [46], [47]], and their encephalitogenic capacity was formally demonstrated in primates [48,49].

MBP is considered a dominating CNS autoantigen but is by no means the only one. Remarkably, MBP-specific CD4^+^ T cells readily mediate EAE in some rodent strains (Lewis rats) but not in others (C57BL mice). In fact, in C57BL mice, myelin-oligodendrocyte glycoprotein (MOG) was identified as the dominant encephalitogen [[50], [51], [52]]. Interestingly, the transfer of MOG-specific T cells into Lewis rats (the strongest responders to MBP immunization) results in only mild clinical EAE [14,53]. Thus, EAE can be induced in different species using various encephalitogenic proteins, but the autoimmune potential of these proteins is strongly species specific. For example, in rabbits, proteolipid protein (PLP) was found to be encephalitogenic [54] and was later used to induce EAE in mice. In addition, MBP induces EAE in guinea pigs [55]. These findings suggest that the stimulation of preexisting myelin-specific T cells with their cognate antigens can disrupt tolerance and induce MS-like CNS inflammation.

Additionally, as summarized in Table 1, each EAE variant has a unique clinical course. However, to date, there are no EAE models that show the complete clinical course of MS. Therefore, researchers need to select an appropriate model depending on the purpose of their scientific questions. In the following sections, we will review the cell cycle of autoreactive T cells, which were mainly studied by using several EAE models, and we will examine the modulatory effect of the gut microbiota on this differentiation.

**Table 1:** Commonly used rodent EAE models.

Brain antigen	Species	Brief description of the model	Clinical outcome or remarks	reference
MBP	SJL/J	Active	Relapse-remitting	[162]
MBP89-169	SJL/J	Transfer	Relapse-remitting	[163]
MBP1-37	PL/J	Active	Relapse	[164]
MBP	PL/J, (PLSJ)F1	Transfer	Relapse (2/3) and chronic (1/3)	[165]
MBP	Lewis rat	Transfer	Monophasic	[4]
PLP	Rabbit	Active	Chronic	[166]
PLP103-116	SWR	Active	Acute	[167]
PLP139-151	SJL/J	Active	Relapse-remitting	[168]
		Transfer		
PLP139-151	SJL/J	Active	Acute	([169,169]
MBP87-99	SJL/J	Active	Relapse	[170]
PLP139-151	SJL/J	Transfer	Relapse-remitting	[170]
MBP84-104 to PLP139-151	SJL/J	Transfer	Epitope spreading	[171]
PLP139-151 to PLP171-191				
MOG35-55 (Th(IgHMOG))	C57BL/6	Spontaneous	Opticospinal EAE	[84]
MOG92-106	SJL/J	Spontaneous	Relapse-remitting	[172]
MOG1-22	Biozzi AB/H	Active	Mild or silent	[50]
MOG43-57				
MOG-134-148				
MOG92-106	SJL/J	Active	Relapse-remitting	[50]
MOG35-55	PL/J	Transfer	Relapse-remitting	[173]
MOG35-55	C57BL/6	Active, transfer	Chronic	[51]
MOG35-55	C3H.SW	Active	Chronic	[51]
MOG(1–121)	C57BL/6	Active	Chronic	[174]
			Involved B cells	
MOG(1–121)	NOD/Lt	Active	Relapse-remitting	
			Involved B cells	
PLP139-151	B10.S	Transfer	EAE resistant	[175]

## Life history of migrant autoreactive T cells in vivo

### Formation in the thymus and the influence of the intestinal environment

Autoimmune T lymphocytes, like all immune competent T cells, retrace their origin to the thymus [56]. They are the progeny of primitive but dedicated precursor cells, which arrive in the organ from the bone marrow via the bloodstream. The immature progenitors enter the thymus at the cortico-medullary interface and then, guided by soluble signals and cell adhesion molecules [57,58], settle within the outer cortex, where they undergo a sequence of developmental changes that ultimately leads to their functional maturity.

The entire maturation process resembles an industrial assembly line, where the differentiating T cell hopeful is forwarded stepwise to topologically coherent but functionally distinct stromal milieus, each of which adds a further trait required to render the T cell immunocompetent [59,60]. This concerns, in particular, the assembly of functional TCR, with the recombination of diverse dimeric receptor chains [61,62]. This is followed by two rounds of positive and negative selection, with the ultimate aim of releasing mature but antigen-unexperienced, naïve T cells into the periphery to be included in the polyclonal immune repertoire of immune cells. Importantly, even in their early phase, immune cells follow the general pattern of their response as young T cells move and recognize the antigen-MHC complex within the thymus.

Intrathymic clonal selection involves interactions with specific local stromal milieu components [57]. In the early phase, which occurs in the thymic cortex, the T cells “learn” to form and expose the double-chained T cell receptor before they enter a positive selection round; exposed to the cortical epithelium, only those T cells that recognize locally presented self-antigens are elected to further proliferate and to proceed to further maturation, while the rest are sorted out, doomed to apoptotic death.

The successfully chosen cells, irrespective of their prospective antigen specificity and function, migrate stepwise toward the thymic medulla before leaving the organ, again via the bloodstream, ultimately settling in peripheral immune tissues. In the medulla, the cells undergo a second, rigorous selection process required to eliminate potentially self-reactive T cells [63,64]. The medullary stroma contains a broad range of marker antigens specific for peripheral organs and tissues. Epithelia expressing the autoimmune regulator gene AIRE^+^ produce and present a broad set of antigens specific for peripheral tissues, including the brain [65]. In addition, there are particular antigen-presenting dendritic cells that enter the thymus and originate from outside important peripheral antigens [66]. Medullary stroma cells present self-antigens as signals to self-reactive T cells, which, following their response, are eliminated.

Negative selection against self-reactive T cells is highly efficient but by no means absolute. Early investigations revealed brain autoreactive CD4^+^ T cell clones circulating in the immune system of healthy rodents [4,44] and humans [45,46,67]. The function of these clones has not been fully elucidated. Certain autoreactive T cells can be beneficial. MBP specific T cells, but not OVA specific T cells, protected neurons from secondary degeneration [68]. In contrast, transfer experiments in rodents demonstrated the potential of these cells to mediate lethal EAE to syngeneic hosts, suggesting that these cells are autoaggressive effector cells. One way that they are kept innocuous throughout the cell cycle may rely on the suppression of peripheral effector T cells (Teff) in their naïve state by a regulatory signaling system (VISTA) [69], a fail-safe system that is lost by inflammatory T cell activation, as it occurs in the gut. On the other hand, at least part of the intrathymic self-reactive population(s) expresses the FoxP3 transcription factor and other markers, indicating that these cells are regulatory T cells (Tregs). It appears that the medullary milieu plays a decisive role in directing mature CD4^+^ T cells toward deletion, naïve Teff or Treg fates. The availability of and responsiveness to IL-2 (via the IL-2 receptor CD25) are known to promote Treg differentiation, with additional support from CD28 costimulation and the regulatory cytokine TGF-β [70].

According to conventional wisdom, the stepwise differentiation of a T cell occurs autonomously in a sterile set of microenvironments, without modulation from the intestine or its microbial content. However, recent observations contradict this simplistic concept. Microbial signaling occurs in the medulla, where the endothelial blood-thymus barrier, which shields the cortex from the outside, is leaky, allowing blood-borne signals to enter the tissue [71].

**Fig. 1:**
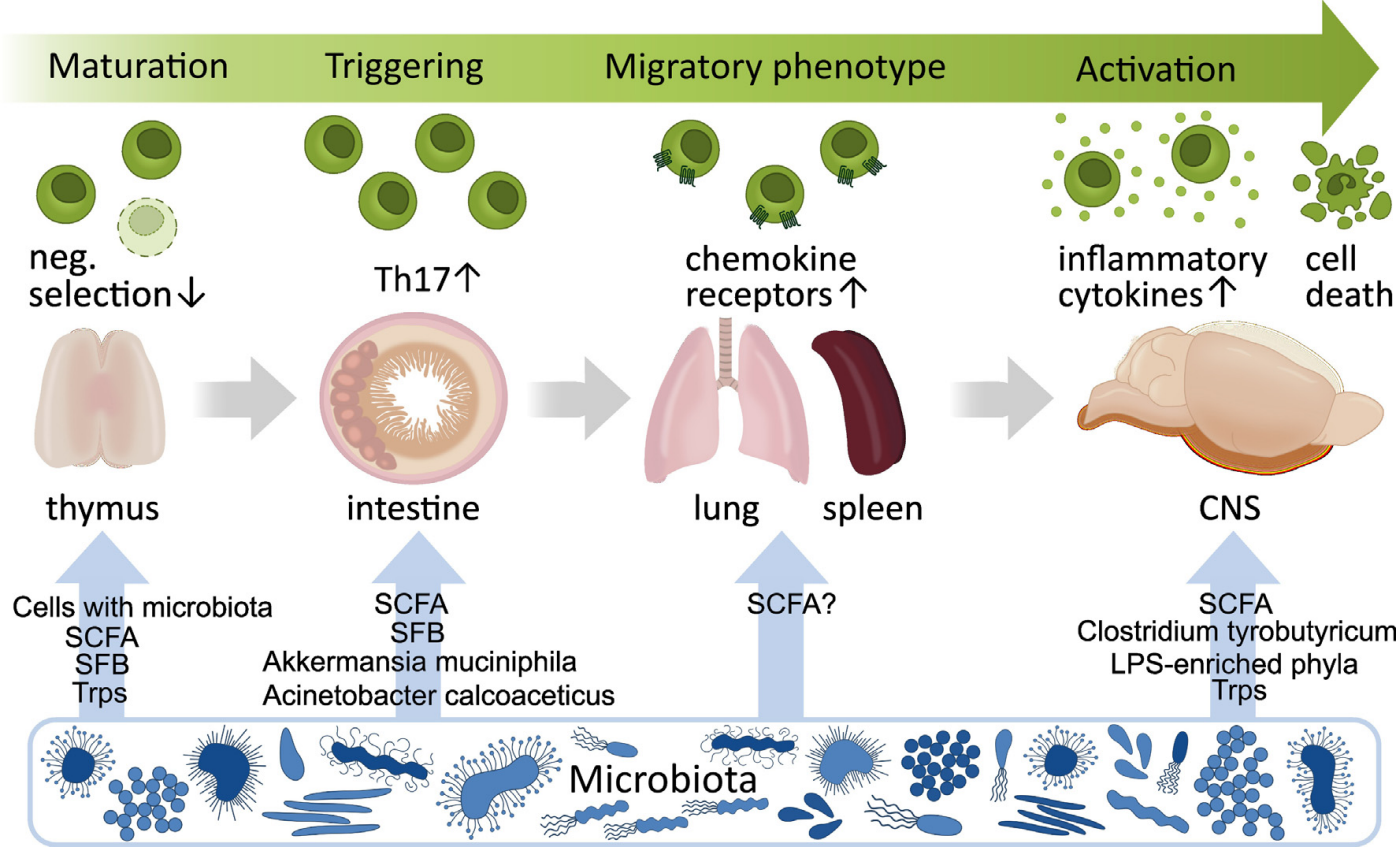
Life of autoreactive T cells under influence of microbiota. Microbiota-induced life events of autoreactive T cells during their journey from the thymus, via the peripheral organs to the CNS are illustrated. SCFA: short-chain fatty acid, SFB: segmented filamentous bacteria, Trps: tryptophan metabolites. Life history of autoreactive T cells under influence of microbiota.

The earliest observations linking the thymus and intestine revealed a growth deficit in the thymus in germ-free mice [72,73]. More recent work confirmed and detailed the microbial impact on thymic function [74]. The gut microbiome emits signals to the thymus essentially via two major channels as soluble compounds in the circulating plasma or contained within circulating myeloid cells (macrophages and classical and plasmacytoid dendritic cells) (Fig. 1). Both enter the target organ through leaky medullary blood vessels, bypassing the cortex. Within the medulla, intestinal messengers act on the maturing T cell population, influencing both the functional lineage decision and the antigen-specific repertoire of immune cells. Soluble gut compounds include bacterial structures that activate local pattern recognition receptors, which, on the medullary epithelium, regulate the expression of AIRE and, implicitly, the deletion of self-reactive T cell clones. Other examples include short-chain fatty acids (SCFAs), metabolic products of dietary fiber digesting colonic bacteria, and tryptophan metabolites (Fig. 1). SCFAs bind to G-coupled receptors on the medullary epithelium to control AIRE-dependent Treg production [75]. An alternative gut-derived signal is segmented filamentous bacteria (SFB)*,* which activates autoimmune Th17 effector cells in the peripheral immune system either by acting through soluble structures reaching the thymus via the bloodstream or being imported by antigen-presenting cells, as noted in SFB-free young mice colonized by SFB (Fig. 1). Intestinal dendritic cells act as vehicles that import SFB antigens into the medulla. The latter route expands microbe-specific T cells in the medulla, which, upon release to the periphery, contribute to the maintenance of antimicrobial tolerance [76]. These observations indicate that the microbiota in the GALT remotely regulates T cell development in the thymus through soluble factors.

### Triggers in the periphery: the role of the gut microbiota

As mentioned, encephalitogenic T cells persist in the healthy repertoire of immune cells without ever causing inflammation in the CNS [47]. However, their pathogenic potential is unleashed upon peripheral activation. Pathogenic triggering may involve adaptive and innate signals, as is the case experimentally with the application of complete Freund's adjuvant (CFA). A more natural trigger is provided by the gut microbiota, as supported by several publications. First, by studying our spontaneous EAE paradigm, we demonstrated that MOG-specific TCR transgenic SJL/J mice, which develop spontaneous EAE under specific pathogen free (SPF) conditions, are largely protected from EAE when kept in a germ-free environment [77]. However, colonization with microbiota derived from SPF mice promptly sparks spontaneous EAE. In addition, we showed that colonization with microbiota derived from MS patients resulted in a significantly greater incidence of disease than colonization with microbiota from healthy control donors [78]. Similarly, we and others reported that the severity of EAE induced by active immunization with MOG/CFA under germ-free conditions was lower than that under SPF conditions [77,79]. Furthermore, the development of EAE is impaired by the oral application of antibiotics, which modify the microbiota [80,81]. These findings highlight the significance of the gut microbiota in the pathogenesis of EAE.

In the spontaneous EAE model, MOG-specific T cells proliferate substantially in the GALT when housed under SPF conditions but much less so in germ-free conditions, indicating microbiota-induced stimulation of T cells [77]. We visualized T cell stimulation in situ via intravital imaging using Twitch, a fluorescent resonant energy transfer (FRET)-based calcium-sensing protein [82,83] (Box 1). We expressed Twitch in MOG-specific T cells prepared from 2D2 transgenic mice [84] or in polyclonal T cells using retroviral gene transfer. The transduced T cells were adoptively transferred to recipient mice and imaged in the GALT using two-photon microscopy. Compared with polyclonal T cells, 2D2 T cells showed greater calcium activity in the ileum, suggesting local stimulation of 2D2 T cells [82]. The stimulation of 2D2 T cells was microbiota- and MHC class II-dependent since calcium activity was reduced in germ-free recipient mice and after the administration of an anti-MHC class II blocking antibody. Interestingly, OVA-specific OTII-T cells and LCMV-specific SMARTA-T cells showed calcium activity similar to that of 2D2 T cells in the ileum, suggesting that T cells are stimulated regardless of their specificity.

Box 1 **Functional fluorescent proteins for intravital imaging of T cells.**

Activation markers (Twitch, Kaede, NFAT, etc.): Structure, application, and examples

Chemical dyes, such as Fura and Indo, can be used to detect changes in the intracellular calcium concentration and are commonly used to detect T cell activation. However, they are not suitable for proliferating cells because they are diluted during cell division. To overcome this limitation, genetically encoded calcium indicators (GECIs), such as GCaMP, were developed to detect cellular activation in vivo. GCaMP changes its signal intensity according to the calcium concentration. This method is effective for stationary cells such as neurons but not for motile cells such as lymphocytes. This is because signal intensity may vary depending on the cell location. For instance, as cells move deeper into tissues or organs, the signal weakens, although the intracellular calcium concentration is unchanged. Therefore, it is necessary to use ratio metric sensors that can compensate for depth-dependent changes in signal intensity when the cells are moving. Twitch2b [83] and Yellow Cameleon [159] are two examples of such sensors. Twitch is composed of cyan fluorescent protein (CFP) and yellow fluorescent protein (YFP), which are linked by the calcium-sensitive troponin C domain. In a low-calcium environment, Twitch emits cyan fluorescence when CFP is excited. However, in a high-calcium environment, the binding of calcium to the troponin C domain alters the protein's structure and causes it to emit yellow fluorescence. Increased intracellular calcium is a hallmark of TCR-mediated stimulation [160] and can be used as a detection method for T cell activation. Twitch can also detect weaker stimulation mediated by chemokine/chemokine receptors in addition to antigen-dependent TCR-mediated stimulation [123]. Additionally, a fluorescent protein-fused activation sensor based on modified nuclear factor of activated T cells (NFAT) is available. The NFAT sensor is a truncated version of NFAT in which the regulatory domain is removed but the DNA binding domain is retained; this sensor is fused with a fluorescent protein. To improve the identification of the location of NFAT within cells, Lodygin et al. used H2B-RFP, which labels the nucleus [132], and we stained the cell body with a chemical dye [133] in combination with an NFAT sensor. Unlike Twitch, the NFAT sensor detects antigen-dependent stimulation, which induces long-lasting calcium stimulation but not weaker stimulation, which is induced by chemokine receptors. Therefore, combining the two sensors can be used to assess the strength of T cell stimulation in vivo.

For in vivo cellular tracking, photoconvertible proteins such as Kaede or Dendra are powerful tools. When exposed to violet light, these proteins change color from green to red, allowing for the tracking of cellular movement between organs. We used lineage-specific Pham transgenic mice expressing Dendra in CD4^+^ T cells to show the migration of T cells from the small intestine to the CNS [82]. Similarly, Kaede can be used for this purpose. Tomura et al. demonstrated the migration kinetics of immune cells from the skin to lymph nodes [161].

We also screened T cell activation in GALT locations outside of the ileal lamina propria [82]. One such location is Peyer's patches, which are lymphoid aggregates located in the submucosa of the small intestine, the major site of intestinal IgA production [85,86]. We imaged 2D2 T cells in Peyer's patches but found no elevated calcium activity, arguing against topical T cell stimulation. This finding is in contrast with that of Song et al., who reported that MBP-specific T cell activation can be observed in Peyer's patches and that these T cells proliferate there after feeding with MBP [87]. These two studies appear conflicting, but their differences seem to reflect the different experimental strategies and methods used. While the imaging experiments portray the real-time response of T cells against homeostatic gut contents, the second study assessed CFSE dye dilution ex vivo to calibrate the T cell response against an orally introduced bolus of foreign protein. Furthermore, we did not observe calcium activity in mesenteric lymph nodes, which drain from the intestine. However, in mesenteric lymph nodes, T cells respond to exogenous antigens provided subcutaneously [88]. The colon can also be a site of T cell activation. Indeed, Duc et al. demonstrated that Th17 ​cells accumulate in the colon, proliferate, and affect the composition of the gut microbiota, implying an interaction between T cells and the microbiota in the colon [89]. In contrast, we did not observe a sufficient number of T cells to analyze calcium activity in the colonic lamina propria. This may be because different T cell types were used. The latter work used Th17-differentiated T cells, while we studied a combination of Th1/Th17 ​T cells. In summary, the ileal lamina propria is the key region where T cells receive stimulation from the microbiota [82]. T cells may also be activated in Peyer's patches and mesenteric lymph nodes under particular experimental conditions but less so in the steady state, most likely due to the lack of antigens.

To document the impact of T cell stimulation in the GALT, we performed a transcriptome analysis of T cells that migrated from the GALT via efferent lymphatic vessels (ductus thoracicus drainage) [82]. Th17-related genes were upregulated in emigrating 2D2 T cells (Fig. 1), which exhibited greater calcium activity in the GALT than did polyclonal T cells that showed lower calcium activity. This may indicate that stimulation in the GALT directs T cells toward the Th17 phenotype. However, in contrast to the findings of Schnell et al. [90], who reported upregulation of the expression of stem-like cell factors, such as SLAMF6, in the GALT, we did not observe this phenotype in lymphatic emigrating T cells. This discrepancy may be because we analyzed the bulk transcriptome, while Schnell et al. relied on single-cell analysis.

In addition to phenotypic changes, we followed the migration of CD4^+^ T cells from the GALT to the CNS in presymptomatic mice using a photoconvertible marker protein (Box 1) [82]. We found that autoreactive T cells do indeed migrate from the GALT to the CNS. However, other migration pathways could not be excluded. For example, Schnell et al. demonstrated that at least some stem-like T cells migrate to the spleen to assist in the shaping of encephalitogenic T cells [90]. Notably, our in vivo tracking experiment confirmed the migration of some CD4^+^ T cells from the GALT to the spleen [82]. Further studies are necessary to reveal the mechanism and routes of T cell migration from the GALT to the CNS.

Some studies were designed to identify the bacteria that contribute to MS/EAE pathogenesis in more detail. Ivanov et al. showed that SFB induced Th17 ​T cells [91], which are considered responsible for EAE initiation [92,93] (Fig. 1). Cekanaviciute et al. demonstrated that the abundances of *Akkermansia muciniphila* and *Acinetobacter calcoaceticus* are greater in the microbiota of people with MS as compared to healthy controls [94] (Fig. 1). In addition, they found that *Akkermansia muciniphila* and *Acinetobacter calcoaceticus* induced a proinflammatory phenotype in human monoclear cells in vitro, as well as in mouse T cells in vivo after the colonization of germ-free mice with these bacteria. We confirmed an increase in Akkermansia abundance in MS patient-derived microbiota compared to healthy controls in our monozygotic twin-pair cohort [78]. In addition to disease-promoting bacteria, disease-suppressing organisms were also identified. For example, the abundance of *Parabacteroides distasonis* was found to be greater in healthy controls than in MS patients [94]. The study also showed that *Parabacteroides distasonis* induced IL-10 production from human T cells in vitro.

The active involvement of the intestine in the pathogenesis of MS appears to be counterintuitive, at least at first sight. However, indirect connections between the gut and CNS are common in clinical neurology. First, people with MS often complain about digestive dysfunctions, but these dysfunctions, for example, constipation and fecal incontinence, are considered secondary consequences of MS-related neurodegeneration [95,96]. Second, there have been countless attempts to mitigate MS-related disability by designing special nutritional formulations, often including unsaturated fatty acids. Much of this was influenced by Swank's classic epidemiologic observation of Norwegian populations, which connected high disease incidence with high-milk diets and lower risk with fish uptake [97]. A pilot trial with small-bowel biopsies revealed subtle changes in some specimens [98] attributed to the failure to absorb fatty acids. Antibiotics were claimed to influence the clinical outcome of MS. Alonso et al. reported that the administration of penicillin was associated with a reduced risk of developing a first attack of MS [99]. More recently, Metz et al. reported that minocycline treatment significantly reduced the risk of conversion from clinically isolated syndrome (CIS) to MS [100]. However, minocycline is reputed to act directly on glia cells apart from affecting gut microbiota [101].

Importantly, fecal microbiota transplantation (FMT) was introduced as a potential treatment for MS. Borody et al. reported the reversal of major neurological symptoms, including an improvement in mobility [102], although this is a case report with only three patients. More detailed study of FMT was recently performed, including an analysis of microbiota compositions and their metabolites, as well as a clinical measurement of the recipients [103], They reported that FMT increased the relative abundance of anti-inflammatory bacteria, improved mobility, and maintained normal gastrointestinal symptoms with no report of relapse during the 12-months study period. Yet, at present, the number of patients included in these FMT studies is relatively limited, and further studies with a larger patient cohort are necessary to gain more comprehensive understanding of the FMT treatment.

There are conflicting reports on whether the microbiota of people with MS differs from that of healthy controls. While some studies, including ours, have found no major differences [78,94], others have reported differences [104]. Takewaki et al. reported alterations in the microbiota at different stages of MS [105]. These discrepancies may be due to differences in the cohorts studied. It is important to note that the microbiota composition can be altered by treatments. For example, the depletion of B cells results in decreased IgA release into the gut lumen. This reduction can impact the composition of the microbiota since IgA plays an important role in shaping the microbial community [106]. As both T cell-dependent and -independent mechanisms control IgA production from B cells [107], T cell-directed therapies can also affect the microbiota through IgA production. In addition, stress [108], genetics and the environment [109] are known to affect the microbiota.

The mechanism by which the microbiota affects T cells is largely unknown. One possible mechanism is molecular mimicry. Due to the large number of microbiota (up to 100 billion organisms [110]), it is not surprising that T cells recognize bacteria-derived antigens as specific targets. As summarized by Garabatos et al., many reports have demonstrated that T cells recognize microbiota-derived peptides [111]. Planas et al. demonstrated that brain-infiltrating CD4^+^ T cells isolated from individuals with MS recognize GDP-l-fucose synthase, which can be derived from bacteria [112]. They also found a significant association between reactivity against GDP-l-fucose synthase and MBP, suggesting an important role of such T cells in MS pathogenesis. In addition, Miyauchi et al. suggested that the microbiota stimulates T cells through two distinct signals [113]. One signal from Erysipelotrichaceae acts as an adjuvant, while the other signal from Lactobacillus provides a peptide that mimics MOG. In line with these results, we demonstrated that T cell stimulation in the GALT is MHC class II dependent, suggesting antigen mimicry [82].

Importantly, in addition to bacterial-derived antigen, viral antigen may also result CNS inflammation. Recently, Bjornevik et al. discovered a tight link between infection with the Epstein-Barr virus (EBV) and the risk of developing MS [114]. Another study demonstrated that antibodies directed toward EBNA1, a nuclear antigen of EBV, exhibited cross-reactivity with glial cell adhesion protein (GlialCAM), which is expressed in the CNS [115]. The precise location of the B cell response, which is responsible for the production of cross-reactive antibodies, remains uncertain. Some findings locate this process within the GALT [116]. Accordingly, EBV infection induces the expression of the matrix binding integrin LPAM-1, integrin α4β7, on memory B cells, which subsequently migrate to GALT, where they receive signals from the microbiota and interact with CD4^+^ T cells.

Another possibility for stimulation is via soluble factors. In this model, short-chain fatty acids (SCFAs) seem to play a crucial role (Fig. 1). SCFAs are fatty acids that contain 2-6 carbon atoms and are produced by microbiota during fermentation. It is not surprising that SCFAs have various effects on different types of cells since they are mixtures of many different compounds. Park et al. demonstrated that SCFAs induce T cell differentiation [117], while Dupraz et al. showed that SCFAs repress IL17 production from γδT cells [118]. According to Trend et al., serum SCFA levels and changes in circulating immune cells are suggested as biomarkers of MS [119]. Finally, it is important to consider signals via Toll-like receptors (TLRs) in relation to MS since TLRs sense the microbiota. The significant roles of TLRs in MS have been discussed for many years, even before the contribution of the microbiota to MS was shown [120]. However, the exact role of TLR signaling is unclear. For example, TLR4-or TLR9-deficient mice showed exaggerated clinical EAE after MOG/CFA, whereas mice lacking MyD88, a common downstream molecule of TLRs, were resistant to EAE in the same model [121]. This could be due to the combination of a large number of potential microbiota-derived ligands and more than 10 different TLRs, which are expressed in different tissues.

In addition to the GALT, we demonstrated that autoreactive T cells can also acquire a migratory phenotype in the spleen [122]. This phenotype is characterized by the upregulation of certain chemokine receptors, which most likely allows T cells to pass through the BBB more efficiently and induce clinical EAE more quickly than in vitro activated T cells. Additionally, we showed that the T cells in the spleen showed short-lasting calcium activity [123]. Although a direct link between calcium signaling and the acquisition of a migratory phenotype is missing, the results suggest that stimulation from the local environment of the spleen induces a migratory phenotype. We showed that chemokine signals contribute to T cell stimulation in the spleen [123], and microbiota-derived metabolites, such as SCFAs, may also contribute. In addition to the spleen, Odoardi et al. demonstrated that T cells can acquire a migratory phenotype in the lung [124]. The lung is an interesting organ, as is the intestine, as both are with their own microbiota and continuously exposed to environmental factors. It is well known that environmental factors play a role in the pathogenesis of MS. For example, smoking, which directly affects the lung, is a well-known risk factor for MS [125].

In summary, the accumulating results clearly document the importance of the microbiota in MS/EAE induction. However, further studies are needed to answer many questions that remain unanswered, such as which bacteria are involved and how they affect specific cells.

## Effector phase in the CNS

After autoreactive T cells reach the CNS vasculature, they attach loosely to endothelial cells and move over their intraluminal surface by rolling and crawling. Crawling T cells preferentially move against the blood flow. Some T cells extravasate through the vascular wall [13]. These interactions, as revealed by intravital imaging with calcium-sensing proteins, did not involve any calcium activation of the T cells [123]. However, these interactions depend on cell adhesion molecules. For example, inhibition of integrin alpha4 diminished intraluminal crawling and prevented the development of clinical EAE [13,126]. This observation is in accordance with the action of the alpha4-binding antibody natalizumab, one of the most effective treatments for MS. Further, a genome-wide screen revealed that in addition to integrin alpha4, CXCR3 and Grk2 regulate the extravasation of T cells from the blood into the CNS [127]. Additional functional structures, including P-selectin [128], LFA1 [129], ninjurin1 [124], and integrin alpha4beta7 [130], also contribute, depending on the phenotype of the T cells and the EAE model used.

Once they extravasate into the leptomeningeal space, encephalitogenic T cells interact with local antigen-presenting cells, such as perivascular macrophages, through extensive calcium signaling that can last up to 2 ​h [123]. Consistently, Othy et al. showed that Th17 ​cells exhibit calcium activity when interacting with APCs in the spinal cord meninges [131]. We found that the stimulation was antigen specific, as only MBP-specific T cells, not OVA-specific T cells, responded to calcium in the leptomeninges [123]. In addition, the administration of anti-MHC class II antibodies significantly reduced calcium activity, indicating that the stimulation depends on the antigen/MHC class II complex. In addition to the Twitch calcium-sensing protein, we and others developed a nuclear factor of activated T cells (NFAT)-based sensor to detect T cell stimulation in vivo (Box 1). This approach visualized the translocation of the NFAT sensor from the cytosol to the nucleus in autoreactive T cells in the CNS after they interacted with local antigen-presenting cells [132,133]. Furthermore, we observed stable interactions between T cells and APCs in the spinal cord parenchyma using live imaging of explants [134], which indicated that T cell activation can be visualized by calcium signaling, as can the translocation of the NFAT sensor [123]. This suggests that infiltrating autoreactive T cells are reactivated by local APCs that present endogenous antigens [13,14].

T-cell reactivation within the CNS is causally related to the pathogenesis of EAE. Calcium activity, i.e., T cell activation, and the clinical severity of EAE are significantly reduced by anti-MHC class II blocking antibodies or small molecular inhibitors of intracellular calcium signaling [123,135]. Conversely, we demonstrated that clinical severity was exacerbated by enhancing T cell activation within the CNS through the administration of exogenous antigens. Specifically, administration of exogenous MOG or S100b protein antigens into the CSF after adoptive transfer of MOG- or S100b-specific T cells extensively increased encephalitogenicity [14]. Similarly, the administration of exogenous MBP after the infiltration of MBP-specific T cells to the CNS significantly exacerbates clinical EAE [136]. Local antigen presentation is not restricted to myelin autoantigens but also involves foreign antigens, such as ovalbumin (OVA). Intrathecal injection of OVA after adoptive transfer of OVA-specific T cells induced the production of inflammatory cytokines by OVA-specific T cells and subsequent body weight loss, indicating that nonpathogenic T cells can be converted to pathogenic T cells [13]. These observations highlight the significance of sequential T cell activation in various locations and suggest that preventing T cell activation at any stage could be a potential therapeutic strategy.

To understand how T cells are activated in the CNS, identifying the responsible APC is crucial. It is important to note that historically, the CNS has been considered an immune-privileged organ [137,138] with severely limited expression of MHC class II antigens on homeostatic CNS resident cells [139]. However, T cells infiltrating the CNS robustly induce MHC class II antigens in microglia [140,141], which allows these glial cells to act as APCs. In addition, we identified CD11b^+^ myeloid cells as potential APCs in the CNS of a Lewis rat model of EAE [13], while others described CD11c^+^ dendritic cells (DCs) [142] and group 3 innate lymphoid cells (ILC3s) [143] as APCs in active EAE mice. Recently, Jordão et al. presented a precise analysis of myeloid cells in the CNS at the single-cell level during neuroinflammation, which provided important information about crucial APCs [144]. This study demonstrated that macrophages undergo rapid proliferation and phenotypic changes during CNS inflammation. In addition, intravital imaging revealed that CCR2^+^ infiltrated macrophages had the highest frequency of long-lasting contact with T cells among the tested myeloid cells. However, CNS-resident macrophages and microglia also exhibited long-lasting contacts, suggesting redundancy of antigen-presenting cells in the CNS.

Together, these findings suggest the following scenario. Activated autoreactive T cells produce inflammatory cytokines and recruit other types of accessory immune cells, such as macrophages, B cells and endogenous T cells [14]. The fate of T cells after the induction of local inflammation is not clear. Some CD8^+^ T cells remain in the CNS as tissue-resident memory T cells in the relapse-remitting EAE model [145] and in MS patients [146]. In the presence of CD4^+^ T cells, these tissue-resident CD8^+^ T cells can expand and induce tissue inflammation [147]. In contrast, less is known about CD4^+^ tissue-resident memory T cells in the CNS. In the Lewis rat EAE model, autoreactive T cells largely disappear from the CNS during the recovery phase of EAE [122,148]. Although the detailed mechanisms of cell clearance are not clear, apoptosis-mediated [149] and FasL-mediated [150] elimination of T cells have been reported. However, some autoreactive T cells become memory T cells and can persist in peripheral organs for their lifetime [148]. These memory T cells can be activated during subsequent inflammation at later time points and efficiently induce clinical EAE.

How do the microbiota and its metabolites affect T cells in the CNS? The microbiota affects the BBB and glial cells through SCFAs, thereby controlling T cell trafficking and activation (Fig. 1). Braniste et al. reported increased BBB permeability in germ-free mice [151]. In addition, colonization with *Clostridium tyrobutyricum* restored BBB integrity (Fig. 1). Although the role of microglia in EAE has not been completely clarified, some studies have shown that they can influence the clinical severity of EAE [152,153]. The microbiota profoundly affects CNS inflammation by influencing the maturation of microglia and their function, as previously shown [154]. A recent study by Hosang et al. demonstrated the mechanism by which the lung microbiota alters the microglial phenotype and impacts EAE [155] (Fig. 1). This work showed that intratracheal neomycin treatment shifted the lung microbiota to lipopolysaccharide-enriched phyla and induced type-I-interferon-primed microglia, which mitigated local inflammation and ameliorated clinical severity. In addition, the intrathecal injection of LPS also reduced the clinical severity of the disease. The production of type-I-interferon from colonic DCs through IFNβ, which was induced by Bacteroidetes, was also shown [156]. Another study by Rothhammer et al. in mice showed that activation of the aryl hydrocarbon receptor (AhR), a receptor for microbiota-derived tryptophan metabolites, exacerbated EAE [157,158] (Fig. 1). This response occurred via decreased TGFα and increased VEGF-B production by microglia, resulting in increased proinflammatory astrocytes. These studies suggest that the microbiota influences resident cells in the CNS and controls the severity of EAE.

We have retraced the life long journey of a prototypical brain autoreactive CD4^+^ T cell from its birth in the thymus to its functional destination within the central nervous parenchyma. Throughout its life, the cell moves from one tissue milieu to the next. At each stopover, the cell receives distinct signals that stepwise instruct the T cell to ultimately home to its programmed target tissue, the brain.

We propose the following scenario:

The journey starts in the thymus, where T cells reach immune competence and where they sneak through self-tolerogenic negative selection. The T cells enter the peripheral blood circulation and briefly settle in peripheral lymphatic organs, including the gut-associated lymphatic tissue (GALT), where they are confronted with the intestinal microbiome. Depending on the bacterial content and the surrounding milieu, T cells may be activated to unleash their autoaggressive potential. They then re-enter the circulation, travel to the central nervous system, cross the endothelial blood‒brain barrier and intrude into the parenchyma. After interacting with local antigen-presenting cells, the cells become reactivated and start a local inflammatory response that, in cooperation with local innate immune cells, results in inflammatory demyelination, the hallmark of human multiple sclerosis.

## Author Contributions

NK and HW wrote manuscript.

## Declaration of competing interest

The authors declare the following financial interests/personal relationships which may be considered as potential competing interests: Naoto Kawakami reports financial support was provided by Deutsche Forschungsgemeinschaft. Hartmut Wekerle reports financial support was provided by International Multiple Sclerosis Microbiome Study. If there are other authors, they declare that they have no known competing financial interests or personal relationships that could have appeared to influence the work reported in this paper.

## References

[1] Bar-Or A. (2008). The immunology of multiple sclerosis. Semin Neurol.

[2] Frischer J.M., Weigand S.D., Guo Y., Kale N., Parisi J.E., Pirko I. (2015). Clinical and pathological insights into the dynamic nature of the white matter multiple sclerosis plaque. Ann Neurol.

[3] Hartung H.P., Cree B.A.C., Barnett M., Meuth S.G., Bar-Or A., Steinman L. (2023). Bioavailable central nervous system disease-modifying therapies for multiple sclerosis. Front Immunol.

[4] Ben-Nun A., Wekerle H., Cohen I.R. (1981). The rapid isolation of clonable antigen-specific T lymphocyte lines capable of mediating autoimmune encephalomyelitis. Eur J Immunol.

[5] Pitarokoili K., Ambrosius B., Gold R. (2017). Lewis rat model of experimental autoimmune encephalomyelitis. Current protocols in neuroscience.

[6] Tanaka Y., Arima Y., Higuchi K., Ohki T., Elfeky M., Ota M. (2017). EAE induction by passive transfer of MOG-specific CD4(+) T cells. Bio Protoc.

[7] Höftberger R., Lassmann H., Berger T., Reindl M. (2022). Pathogenic autoantibodies in multiple sclerosis — from a simple idea to a complex concept. Nat Rev Neurol.

[8] Parodi B., Kerlero de Rosbo N. (2021). The gut-brain Axis in multiple sclerosis. Is its dysfunction a pathological trigger or a consequence of the disease?. Front Immunol.

[9] Mowat A.M., Agace W.W. (2014). Regional specialization within the intestinal immune system. Nat Rev Immunol.

[10] Zheng D., Liwinski T., Elinav E. (2020). Interaction between microbiota and immunity in health and disease. Cell Res.

[11] Louveau A., Harris T.H., Kipnis J. (2015). Revisiting the mechanisms of CNS immune privilege. Trends Immunol.

[12] Louveau A., Smirnov I., Keyes T.J., Eccles J.D., Rouhani S.J., Peske J.D. (2015). Structural and functional features of central nervous system lymphatic vessels. Nature.

[13] Bartholomäus I., Kawakami N., Odoardi F., Schläger C., Miljkovic D., Ellwart J.W. (2009). Effector T cell interactions with meningeal vascular structures in nascent autoimmune CNS lesions. Nature.

[14] Kawakami N., Lassmann S., Li Z., Odoardi F., Ritter T., Ziemssen T. (2004). The activation status of neuroantigen-specific T cells in the target organ determines the clinical outcome of autoimmune encephalomyelitis. J Exp Med.

[15] Madsen L.S., Andersson E.C., Jansson L., Krogsgaard M., Andersen C.B., Engsberg J. (1999). A humanized model for multiple sclerosis using HLA DR2 and a human T cell receptor. Nat Genet.

[16] Jäger A., Dardalhon V., Sobel R.A., Bettelli E., Kuchroo V.K. (2009). Th1, Th17, and Th9 effector cells induce experimental autoimmune encephalomyelitis with different pathological phenotypes. J Immunol.

[17] Hafler D.A., Compston A., Sawcer S., Lander E.S., Daly M.J., De Jager P.L., C. The International Multiple Sclerosis Genetics (2007). Risk alleles for multiple sclerosis identified by a genomewide study. N Engl J Med.

[18] Patsopoulos N.A., Baranzini S.E., Santaniello A., Shoostari P., Cotsapas C., Wong G., Consortium∗†, I.M.S.G. (2019). Multiple sclerosis genomic map implicates peripheral immune cells and microglia in susceptibility. Science.

[19] Gilhus N.E., Skeie G.O., Romi F., Lazaridis K., Zisimopoulou P., Tzartos S. (2016). Myasthenia gravis - autoantibody characteristics and their implications for therapy. Nat Rev Neurol.

[20] Prüss H. (2021). Autoantibodies in neurological disease. Nat Rev Immunol.

[21] Obermeier B., Mentele R., Malotka J., Kellermann J., Wekerle H., Lottspeich F. (2008). Matching of oligoclonal Ig transcriptomes and proteomes of cerebrospinal fluid in multiple sclerosis. Nat Med.

[22] Brändle S.M., Obermeier B., Senel M., Bruder J., Mentele R., Khademi M. (2016). Distinct oligoclonal band antibodies in multiple sclerosis recognize ubiquitous self-proteins. Proc Natl Acad Sci USA.

[23] Lennon V.A., Wingerchuk D.M., Kryzer T.J., Pittock S.J., Lucchinetti C.F., Fujihara K. (2004). A serum autoantibody marker of neuromyelitis optica: distinction from multiple sclerosis. Lancet.

[24] Traub J., Häusser-Kinzel S., Weber M.S. (2020). Differential effects of MS therapeutics on B cells-implications for their use and failure in AQP4-positive NMOSD patients. Int J Mol Sci.

[25] Bradl M., Misu T., Takahashi T., Watanabe M., Mader S., Reindl M. (2009). Neuromyelitis optica: pathogenicity of patient immunoglobulin in vivo. Ann Neurol.

[26] Wright S.K., Wassmer E., Vincent A. (2021). Pathogenic antibodies to AQP4: neuromyelitis optica spectrum disorder (NMOSD). Biochim Biophys Acta Biomembr.

[27] Thomas O.G., Bronge M., Tengvall K., Akpinar B., Nilsson O.B., Holmgren E. (2023). Cross-reactive EBNA1 immunity targets alpha-crystallin B and is associated with multiple sclerosis. Sci Adv.

[28] Pugliese A. (2017). Autoreactive T cells in type 1 diabetes. J Clin Invest.

[29] Zhang F., Wei K., Slowikowski K., Fonseka C.Y., Rao D.A., Kelly S. (2019). Defining inflammatory cell states in rheumatoid arthritis joint synovial tissues by integrating single-cell transcriptomics and mass cytometry. Nat Immunol.

[30] Kaskow B.J., Baecher-Allan C. (2018). Effector T cells in multiple sclerosis. Cold Spring Harbor perspectives in medicine.

[31] Sospedra M., Martin R. (2005). Immunology of multiple sclerosis. Annu Rev Immunol.

[32] Bielekova B., Sung M.H., Kadom N., Simon R., McFarland H., Martin R. (2004). Expansion and functional relevance of high-avidity myelin-specific CD4 + T cells in multiple sclerosis. J Immunol.

[33] Cao Y., Goods B.A., Raddassi K., Nepom G.T., Kwok W.W., Love J.C. (2015). Functional inflammatory profiles distinguish myelin-reactive T cells from patients with multiple sclerosis. Sci Transl Med.

[34] Hellings N., Baree M., Verhoeven C., D'Hooghe M.B., Medaer R., Bernard C.C.A. (2001). T-cell reactivity to multiple myelin antigens in multiple sclerosis patients and healthy controls. J Neurosci Res.

[35] Spadaro M., Winklmeier S., Beltran E., Macrini C., Hoftberger R., Schuh E. (2018). Pathogenicity of human antibodies against myelin oligodendrocyte glycoprotein. Ann Neurol.

[36] Flach A.C., Litke T., Strauss J., Haberl M., Gomez C.C., Reindl M. (2016). Autoantibody-boosted T-cell reactivation in the target organ triggers manifestation of autoimmune CNS disease. Proc Natl Acad Sci USA.

[37] Beltrán E., Paunovic M., Gebert D., Cesur E., Jeitler M., Höftberger R. (2021). Archeological neuroimmunology: resurrection of a pathogenic immune response from a historical case sheds light on human autoimmune encephalomyelitis and multiple sclerosis. Acta Neuropathol.

[38] Súkeníková L., Mallone A., Schreiner B., Ripellino P., Nilsson J., Stoffel M. (2024). Autoreactive T cells target peripheral nerves in Guillain-Barré syndrome. Nature.

[39] Ben-Nun A., Kaushansky N., Kawakami N., Krishnamoorthy G., Berer K., Liblau R. (2014). From classic to spontaneous and humanized models of multiple sclerosis: impact on understanding pathogenesis and drug development. J Autoimmun.

[40] Wekerle H., Flügel A., Fugger L., Schett G., Serreze D.V. (2012). Autoimmunity's next top models. Nat Med.

[41] Rivers T.M., Sprunt D.H., Berry G.P. (1933). Observations on attempts to produce acute disseminated encephalomyelitis in monkeys. J Exp Med.

[42] Kies M.W., Alvord E.C., Roboz E. (1958). Production of experimental allergic encephalomyelitis in Guinea pigs with fractions isolated from bovine spinal cord and killed tubercle bacilli. Nature.

[43] Ben-Nun A., Eisenstein S., Cohen I.R. (1982). Experimental autoimmune encephalomyelitis (EAE) in genetically resistant rats: PVG rats resist active induction of EAE but are susceptible to and can generate EAE effector T cell lines. J Immunol.

[44] Schluesener H.J., Wekerle H. (1985). Autoaggressive T lymphocyte lines recognizing the encephalitogenic region of myelin basic protein: in vitro selection from unprimed rat T lymphocyte populations. J Immunol.

[45] Martin R., Jaraquemada D., Flerlage M., Richert J.R., Whitaker J., Long E.O. (1990). Fine specificity and HLA restriction of myelin basic protein- specific cytotoxic T cell lines from multiple sclerosis patients and healthy individuals. J Immunol.

[46] Ota K., Matsui M., Milford E.L., Mackin G.A., Weiner H.L., Hafler D.A. (1990). T-cell recognition of an immunodominant myelin basic protein epitope in multiple sclerosis. Nature.

[47] Pette M., Fujita K., Kitze B., Whitaker J.N., Albert E., Kappos L. (1990). Myelin basic protein-specific T lymphocyte lines from MS patients and healthy individuals. Neurology.

[48] Genain C.P., Lee-Parritz D., Nguyen M.H., Massacesi L., Joshi N., Ferrante R. (1994). In healthy primates, circulating autoreactive T cells mediate autoimmune disease. J Clin Invest.

[49] Meinl E., Hoch R.M., Dornmair K., De Waal Malefijt R., Bontrop R.E., Jonker M. (1997). Encephalitogenic potential of myelin basic protein-specific T cells isolated from normal rhesus macaques. Am J Pathol.

[50] Amor S., Groome N., Linington C., Morris M.M., Dornmair K., Gardinier M.V. (1994). Identification of epitopes of myelin oligodendrocyte glycoprotein for the induction of experimental allergic encephalomyelitis in SJL and Biozzi AB/H mice. J Immunol.

[51] Mendel I., Kerlero de Rosbo N., Ben-Nun A. (1995). A myelin oligodendrocyte glycoprotein peptide induces typical chronic experimental autoimmune encephalomyelitis in H-2 b mice: fine specificity and T cell receptor Vá expression of encephalitogenic T cells. Eur J Immunol.

[52] Voskuhl R.R., MacKenzie-Graham A. (2022). Chronic experimental autoimmune encephalomyelitis is an excellent model to study neuroaxonal degeneration in multiple sclerosis. Front Mol Neurosci.

[53] Mader S., Ho S., Wong H.K., Baier S., Winklmeier S., Riemer C. (2023). Dissection of complement and Fc-receptor-mediated pathomechanisms of autoantibodies to myelin oligodendrocyte glycoprotein. Proc Natl Acad Sci USA.

[54] Waksman B.H., Porter H., Lees M.B., Adams R.D., Folch J. (1954). A study of the chemical nature of components of bovine white matter effective in producing allergic encephalomyelitis in the rabbit. J Exp Med.

[55] Roboz-Einstein E., Robertson D., DiCaprio J.M., Moore W. (1962). The isolation from bovine spinal cord of homogeneous protein with encephalitogenic activity. J Neurochem.

[56] Ashby K.M., Hogquist K.A. (2024). A guide to thymic selection of T cells. Nat Rev Immunol.

[57] Irla M. (2022). Instructive cues of thymic T cell selection. Annu Rev Immunol.

[58] Kawakami N., Nishizawa F., Sakane N., Iwao M., Tsujikawa K., Ikawa M. (1999). Roles of integrins and CD44 on the adhesion and migration of fetal liver cells to the fetal thymus. J Immunol.

[59] Baldwin I., Robey E.A. (2024). Adjusting to self in the thymus: CD4 versus CD8 lineage commitment and regulatory T cell development. J Exp Med.

[60] Klein L., Kyewski B., Allen P.M., Hogquist K.A. (2014). Positive and negative selection of the T cell repertoire: what thymocytes see (and don't see). Nat Rev Immunol.

[61] Davis M.M., Bjorkman P.J. (1988). T-cell antigen receptor genes and T-cell recognition. Nature.

[62] Jackson K.J., Kidd M.J., Wang Y., Collins A.M. (2013). The shape of the lymphocyte receptor repertoire: lessons from the B cell receptor. Front Immunol.

[63] Klein L., Hinterberger M., Wirnsberger G., Kyewski B. (2009). Antigen presentation in the thymus for positive selection and central tolerance induction. Nat Rev Immunol.

[64] Marx A., Yamada Y., Simon-Keller K., Schalke B., Willcox N., Ströbel P. (2021). Thymus and autoimmunity. Semin Immunopathol.

[65] Takaba H., Takayanagi H. (2017). The mechanisms of T cell selection in the thymus. Trends Immunol.

[66] Perry J.S.A., Lio C.W.J., Kau A.L., Nutsch K., Yang Z., Gordon J.I. (2014). Distinct contributions of Aire and antigen-presenting-cell subsets to the generation of self-tolerance in the thymus. Immunity.

[67] Pette M., Fujita K., Wilkinson D., Altmann D.M., Trowsdale J., Giegerich G. (1990). Myelin autoreactivity in multiple sclerosis: recognition of myelin basic protein in the context of HLA-DR2 products by T lymphocytes of multiple sclerosis patients and healthy donors. Proc Natl Acad Sci USA.

[68] Moalem G., Leibowitz-Amit R., Yoles E., Mor F., Cohen I.R., Schwartz M. (1999). Autoimmune T cells protect neurons from secondary degeneration after central nervous system axotomy. Nat Med.

[69] ElTanbouly M.A., Zhao Y., Nowak E., Li J., Schaafsma E., Le Mercier I. (2020). VISTA is a checkpoint regulator for naïve T cell quiescence and peripheral tolerance. Science.

[70] Klein L., Robey E.A., Hsieh C.S. (2019). Central CD4 + T cell tolerance: deletion versus regulatory T cell differentia tion. Nat Rev Immunol.

[71] Raviola E., Karnovsky M.J. (1972). Evidence for a blood-thymus barrier using electron-opaque tracers. J Exp Med.

[72] Bealmear M., Wilson R. (1966). Histological comparison of the thymus of germfree (axenic) and conventional CFW mice. Anat Rec.

[73] Wilson R., Bealmear M., Sobonya R. (1965). Growth and regressionof the germfree (axenic) thymus. Proc Soc Exp Biol Med.

[74] Hebbandi Nanjundappa R., Sokke Umeshappa C., Geuking M.B. (2022). The impact of the gut microbiota on T cell ontogeny in the thymus. Cell Mol Life Sci : CM.

[75] Ni D., Tan J., Robert R., Taitz J., Ge A., Potier-Villette C. (2023). GPR109A expressed on medullary thymic epithelial cells affects thymic Treg development. Eur J Immunol.

[76] Zegarra-Ruiz D.F., Kim D.V., Norwood K., Kim M., Wu W.J.H., Saldana-Morales F.B. (2021). Thymic development of gut-microbiota-specific T cells. Nature.

[77] Berer K., Mues M., Koutrolos M., Al Rasbi Z., Boziki M., Johner C. (2011). Commensal microbiota and myelin autoantigen cooperate to trigger autoimmune demyelination. Nature.

[78] Berer K., Gerdes L.A., Cekanaviciute E., Jia X., Xiao L., Xia Z. (2017). Gut microbiota from multiple sclerosis patients enables spontaneous autoimmune encephalomyelitis in mice. Proc Natl Acad Sci USA.

[79] Lee Y.K., Menezes J.S., Umesaki Y., Mazmanian S.K. (2010).

[80] Gödel C., Kunkel B., Kashani A., Lassmann H., Arumugam M., Krishnamoorthy G. (2020). Perturbation of gut microbiota decreases susceptibility but does not modulate ongoing autoimmune neurological disease. J Neuroinflammation.

[81] Ochoa-Repáraz J., Mielcarz D.W., Ditrio L.E., Burroughs A.R., Foureau D.M., Haque-Begum S. (2009). Role of gut commensal microflora in the development of experimental autoimmune encephalomyelitis. J Immunol.

[82] Bauer I.J., Fang P., Lämmle K.F., Tyystjärvi S., Alterauge D., Baumjohann D. (2023). Visualizing the activation of encephalitogenic T cells in the ileal lamina propria by in vivo two-photon imaging. Proc Natl Acad Sci USA.

[83] Thestrup T., Litzlbauer J., Bartholomaus I., Mues M., Russo L., Dana H. (2014). Optimized ratiometric calcium sensors for functional in vivo imaging of neurons and T lymphocytes. Nat Methods.

[84] Bettelli E., Pagany M., Weiner H.L., Linington C., Sobel R.A., Kuchroo V.K. (2003). Myelin oligodendrocyte glycoprotein-specific T cell receptor transgenic mice develop spontaneous autoimmune optic neuritis. J Exp Med.

[85] Mowat A.M. (2003). Anatomical basis of tolerance and immunity to intestinal antigens. Nat Rev Immunol.

[86] Park J.I., Cho S.W., Kang J.H., Park T.E. (2023). Intestinal peyer's patches: structure, function, and in vitro modeling. Tissue engineering and regenerative medicine.

[87] Song F., Wardrop R.M., Gienapp I.E., Stuckman S.S., Meyer A.L., Shawler T. (2008). The Peyer's patch is a critical immunoregulatory site for mucosal tolerance in experimental autoimmune encephalomylelitis (EAE). J Autoimmun.

[88] Hiltensperger M., Beltrán E., Kant R., Tyystjärvi S., Lepennetier G., Domínguez Moreno H. (2021). Skin and gut imprinted helper T cell subsets exhibit distinct functional phenotypes in central nervous system autoimmunity. Nat Immunol.

[89] Duc D., Vigne S., Bernier-Latmani J., Yersin Y., Ruiz F., Gaia N. (2019). Disrupting myelin-specific Th17 cell gut homing confers protection in an adoptive transfer experimental autoimmune encephalomyelitis. Cell Rep.

[90] Schnell A., Huang L., Singer M., Singaraju A., Barilla R.M., Regan B.M.L. (2021). Stem-like intestinal Th17 cells give rise to pathogenic effector T cells during autoimmunity. Cell.

[91] Ivanov I.I., Atarashi K., Manel N., Brodie F.L., Shima T., Karaoz U. (2009). Induction of intestinal Th17 cells by segmented filamentous bacteria. Cell.

[92] Cua D.J., Sherlock J., Chen Y., Murphy C.A., Joyce B., Seymour B. (2003). Interleukin-23 rather than interleukin-12 is the critical cytokine for autoimmune inflammation of the brain. Nature.

[93] Krishnarajah S., Becher B. (2022). T(H) cells and cytokines in encephalitogenic disorders. Front Immunol.

[94] Cekanaviciute E., Yoo B.B., Runia T.F., Debelius J.W., Singh S., Nelson C.A. (2017). Gut bacteria from multiple sclerosis patients modulate human T cells and exacerbate symptoms in mouse models. Proc Natl Acad Sci USA.

[95] Preziosi G., Gordon-Dixon A., Emmanuel A. (2018). Neurogenic bowel dysfunction in patients with multiple sclerosis: prevalence, impact, and management strategies. Degener Neurol Neuromuscul Dis.

[96] Wiesel P.H., Norton C., Glickman S., Kamm M.A. (2001). Pathophysiology and management of bowel dysfunction in multiple sclerosis. Eur J Gastroenterol Hepatol.

[97] Swank R.L., Lerstad O., Strom A., Backer J. (1952). Multiple sclerosis in rural Norway - its geographic and occupational incidence in relation to nutrition. N Engl J Med.

[98] Lange L.S., Shiner M. (1976). Small bowel abnormalities in multiple sclerosis. Lancet.

[99] Alonso A., Jick S.S., Jick H., Hernan M.A. (2006). Antibiotic use and risk of multiple sclerosis. Am J Epidemiol.

[100] Metz L.M., Li D.K.B., Traboulsee A.L., Duquette P., Eliasziw M., Cerchiaro G. (2017). Trial of minocycline in a clinically isolated syndrome of multiple sclerosis. N Engl J Med.

[101] Collongues N., Becker G., Jolivel V., Ayme-Dietrich E., de Seze J., Binamé F. (2022). A narrative review on axonal neuroprotection in multiple sclerosis. Neurology and Therapy.

[102] Borody T., Leis S., Campbell J., Torres M., Nowak A. (2011). Fecal microbiota transplantation (FMT) in multiple sclerosis (MS): 942. Official journal of the American College of Gastroenterology | ACG.

[103] Engen P.A., Zaferiou A., Rasmussen H., Naqib A., Green S.J., Fogg L.F. (2020). Single-arm, non-randomized, time series, single-subject study of fecal microbiota transplantation in multiple sclerosis. Front Neurol.

[104] Chen J., Chia N., Kalari K.R., Yao J.Z., Novotna M., Soldan M.M. (2016). Multiple sclerosis patients have a distinct gut microbiota compared to healthy controls. Sci Rep.

[105] Takewaki D., Yamamura T. (2021). Gut microbiome research in multiple sclerosis. Neurosci Res.

[106] Sutherland D.B., Suzuki K., Fagarasan S. (2016). Fostering of advanced mutualism with gut microbiota by Immunoglobulin A. Immunol Rev.

[107] Fagarasan S., Kawamoto S., Kanagawa O., Suzuki K. (2010). Adaptive immune regulation in the gut: T cell-dependent and T cell-independent IgA synthesis. Annu Rev Immunol.

[108] Kraimi N., Lormant F., Calandreau L., Kempf F., Zemb O., Lemarchand J. (2022). Microbiota and stress: a loop that impacts memory. Psychoneuroendocrinology.

[109] Hall A.B., Tolonen A.C., Xavier R.J. (2017). Human genetic variation and the gut microbiome in disease. Nat Rev Genet.

[110] Hou K., Wu Z.-X., Chen X.-Y., Wang J.-Q., Zhang D., Xiao C. (2022). Microbiota in health and diseases. Signal Transduct Targeted Ther.

[111] Garabatos N., Santamaria P. (2022). Gut microbial antigenic mimicry in autoimmunity. Front Immunol.

[112] Planas R., Santos R., Tomas-Ojer P., Cruciani C., Lutterotti A., Faigle W. (2018). GDP-l-fucose synthase is a CD4(+) T cell-specific autoantigen in DRB3∗02:02 patients with multiple sclerosis. Sci Transl Med.

[113] Miyauchi E., Kim S.-W., Suda W., Kawasumi M., Onawa S., Taguchi-Atarashi N. (2020). Gut microorganisms act together to exacerbate inflammation in spinal cords. Nature.

[114] Bjornevik K., Cortese M., Healy B.C., Kuhle J., Mina M.J., Leng Y. (2022). Longitudinal analysis reveals high prevalence of Epstein-Barr virus associated with multiple sclerosis. Science.

[115] Lanz T.V., Brewer R.C., Ho P.P., Moon J.-S., Jude K.M., Fernandez D. (2022). Clonally expanded B cells in multiple sclerosis bind EBV EBNA1 and GlialCAM. Nature.

[116] Leffler J., Trend S., Hart P.H., French M.A. (2022). Epstein-Barr virus infection, B-cell dysfunction and other risk factors converge in gut-associated lymphoid tissue to drive the immunopathogenesis of multiple sclerosis: a hypothesis. Clinical & translational immunology.

[117] Park J., Kim M., Kang S.G., Jannasch A.H., Cooper B., Patterson J. (2015). Short-chain fatty acids induce both effector and regulatory T cells by suppression of histone deacetylases and regulation of the mTOR–S6K pathway. Mucosal Immunol.

[118] Dupraz L., Magniez A., Rolhion N., Richard M.L., Da Costa G., Touch S. (2021). Gut microbiota-derived short-chain fatty acids regulate IL-17 production by mouse and human intestinal γδ T cells. Cell Rep.

[119] Trend S., Leffler J., Jones A.P., Cha L., Gorman S., Brown D.A. (2021). Associations of serum short-chain fatty acids with circulating immune cells and serum biomarkers in patients with multiple sclerosis. Sci Rep.

[120] Racke M.K., Drew P.D. (2009). Toll-like receptors in multiple sclerosis. Curr Top Microbiol Immunol.

[121] Marta M., Andersson Å., Isaksson M., Kämpe O., Lobell A. (2008). Unexpected regulatory roles of TLR4 and TLR9 in experimental autoimmune encephalomyelitis. Eur J Immunol.

[122] Flügel A., Berkowicz T., Ritter T., Labeur M., Jenne D., Li Z. (2001). Migratory activity and functional changes of green fluorescent effector T cells before and during experimental autoimmune encephalomyelitis. Immunity.

[123] Kyratsous N.I., Bauer I.J., Zhang G., Pesic M., Bartholomaus I., Mues M. (2017). Visualizing context-dependent calcium signaling in encephalitogenic T cells in vivo by two-photon microscopy. Proc Natl Acad Sci USA.

[124] Odoardi F., Sie C., Streyl K., Ulaganathan V.K., Schläger C., Lodygin D. (2012). T cells become licensed in the lung to enter the central nervous system. Nature.

[125] Rodgers J., Friede T., Vonberg F.W., Constantinescu C.S., Coles A., Chataway J. (2021). The impact of smoking cessation on multiple sclerosis disease progression. Brain.

[126] Yednock T.A., Cannon C., Fritz L.C., Sánchez-Madrid F., Steinman L., Karin N. (1992). Prevention of experimental autoimmune encephalomyelitis by antibodies against α4ß1 integrin. Nature.

[127] Kendirli A., de la Rosa C., Lämmle K.F., Eglseer K., Bauer I.J., Kavaka V. (2023). A genome-wide in vivo CRISPR screen identifies essential regulators of T cell migration to the CNS in a multiple sclerosis model. Nat Neurosci.

[128] Sathiyanadan K., Coisne C., Enzmann G., Deutsch U., Engelhardt B. (2014). PSGL-1 and E/P-selectins are essential for T-cell rolling in inflamed CNS microvessels but dispensable for initiation of EAE. Eur J Immunol.

[129] Dusi S., Angiari S., Pietronigro E.C., Lopez N., Angelini G., Zenaro E. (2019). LFA-1 controls Th1 and Th17 motility behavior in the inflamed central nervous system. Front Immunol.

[130] Rossi B., Dusi S., Angelini G., Bani A., Lopez N., Della Bianca V. (2023). Alpha4 beta7 integrin controls Th17 cell trafficking in the spinal cord leptomeninges during experimental autoimmune encephalomyelitis. Front Immunol.

[131] Othy S., Jairaman A., Dynes J.L., Dong T.X., Tune C., Yeromin A.V. (2020). Regulatory T cells suppress Th17 cell Ca(2+) signaling in the spinal cord during murine autoimmune neuroinflammation. Proc Natl Acad Sci USA.

[132] Lodygin D., Odoardi F., Schlager C., Korner H., Kitz A., Nosov M. (2013). A combination of fluorescent NFAT and H2B sensors uncovers dynamics of T cell activation in real time during CNS autoimmunity. Nat Med.

[133] Pesic M., Bartholomaus I., Kyratsous N.I., Heissmeyer V., Wekerle H., Kawakami N. (2013). 2-photon imaging of phagocyte-mediated T cell activation in the CNS. J Clin Invest.

[134] Kawakami N., Nägerl U.V., Odoardi F., Bonhoeffer T., Wekerle H., Flügel A. (2005). Live imaging of effector cell trafficking and autoantigen recognition within the unfolding autoimmune encephalomyelitis lesion. J Exp Med.

[135] Cordiglieri C., Odoardi F., Zhang B., Nebel M., Kawakami N., Klinkert W.E.F. (2010). Nicotinic acid adenine dinucleotide phosphate-mediated calcium signalling in effector T cells regulates autoimmunity of the central nervous system. Brain.

[136] Odoardi F., Kawakami N., Klinkert W.E.F., Wekerle H., Flügel A. (2007). Blood-borne soluble protein antigen intensifies T cell activation in autoimmune CNS lesions and exacerbates clinical disease. Proc Natl Acad Sci USA.

[137] Galea I., Bechmann I., Perry V.H. (2007). What is immune privilege (not)?. Trends Immunol.

[138] Medawar P.B. (1948). Immunity to homologous grafted skin. III. The fate of skin homografts transplanted to the brain, to subcutaneous tissue, and to anterior chamber of the eye. Br J Exp Pathol.

[139] Shrikant P., Benveniste E.N. (1996). The central nervous system as an immunocompetent organ. Role of glial cells in antigen presentation. J Immunol.

[140] McCombe P.A., De Jersey J., Pender M.P. (1994). Inflammatory cells, microglia and MHC class II antigen-positive cells in the spinal cord of Lewis rats with acute and chronic relapsing experimental autoimmune encephalomyelitis. J Neuroimmunol.

[141] Subbarayan M.S., Hudson C., Moss L.D., Nash K.R., Bickford P.C. (2020). T cell infiltration and upregulation of MHCII in microglia leads to accelerated neuronal loss in an α-synuclein rat model of Parkinson's disease. J Neuroinflammation.

[142] Greter M., Heppner F.L., Lemos M.P., Odermatt B., Goebels N., Laufer T.M. (2005). Dendritic cells permit immune invasion of the CNS in an animal model of multiple sclerosis. Nat Med.

[143] Grigg J.B., Shanmugavadivu A., Regen T., Parkhurst C.N., Ahmed A., Joseph A.M. (2021). Antigen-presenting innate lymphoid cells orchestrate neuroinflammation. Nature.

[144] Jordao M.J.C., Sankowski R., Brendecke S.M., Sagar, Locatelli G., Tai Y.H. (2019). Single-cell profiling identifies myeloid cell subsets with distinct fates during neuroinflammation. Science.

[145] Sasaki K., Bean A., Shah S., Schutten E., Huseby P.G., Peters B. (2014). Relapsing-remitting central nervous system autoimmunity mediated by GFAP-specific CD8 T cells. J Immunol.

[146] Machado-Santos J., Saji E., Tröscher A.R., Paunovic M., Liblau R., Gabriely G. (2018). The compartmentalized inflammatory response in the multiple sclerosis brain is composed of tissue-resident CD8+ T lymphocytes and B cells. Brain.

[147] Vincenti I., Page N., Steinbach K., Yermanos A., Lemeille S., Nunez N. (2022). Tissue-resident memory CD8^+^ T cells cooperate with CD4^+^ T cells to drive compartmentalized immunopathology in the CNS. Sci Transl Med.

[148] Kawakami N., Odoardi F., Ziemssen T., Bradl M., Ritter T., Neuhaus O. (2005). Autoimmune CD4 + T cell memory: lifelong persistence of encephalitogenic T cell clones in healthy immune repertoires. J Immunol.

[149] Bauer J., Bradl M., Hickey W.F., Forss-Petter S.J., Breitschopf H., Linington C. (1998). T cell apoptosis in inflammatory brain lesions. Destruction of T cells does not depend on antigen recognition. Am J Pathol.

[150] Medana I., Li Z.X., Flügel A., Tschopp J., Wekerle H., Neumann H. (2001). Fas ligand (CD95L) protects neurons against perforin-mediated T lymphocyte cytotoxicity. J Immunol.

[151] Braniste V., Al-Asmakh M., Kowal C., Anuar F., Abbaspour A., Tóth M. (2014). The gut microbiota influences blood-brain barrier permeability in mice. Sci Transl Med.

[152] Jie Z., Ko C.J., Wang H., Xie X., Li Y., Gu M. (2021). Microglia promote autoimmune inflammation via the noncanonical NF-κB pathway. Sci Adv.

[153] Montilla A., Zabala A., Er-Lukowiak M., Rissiek B., Magnus T., Rodriguez-Iglesias N. (2023). Microglia and meningeal macrophages depletion delays the onset of experimental autoimmune encephalomyelitis. Cell Death Dis.

[154] Erny D., Hrabě de Angelis A.L., Jaitin D., Wieghofer P., Staszewski O., David E. (2015). Host microbiota constantly control maturation and function of microglia in the CNS. Nat Neurosci.

[155] Hosang L., Canals R.C., van der Flier F.J., Hollensteiner J., Daniel R., Flügel A. (2022). The lung microbiome regulates brain autoimmunity. Nature.

[156] Stefan K.L., Kim M.V., Iwasaki A., Kasper D.L. (2020). Commensal microbiota modulation of natural resistance to virus infection. Cell.

[157] Rothhammer V., Borucki D.M., Tjon E.C., Takenaka M.C., Chao C.-C., Ardura-Fabregat A. (2018). Microglial control of astrocytes in response to microbial metabolites. Nature.

[158] Rothhammer V., Quintana F.J. (2019). The aryl hydrocarbon receptor: an environmental sensor integrating immune responses in health and disease. Nat Rev Immunol.

[159] Jin K., Imada T., Nakamura S., Izuta Y., Oonishi E., Shibuya M. (2018). Intravital two-photon imaging of Ca(2+) signaling in secretory organs of yellow Cameleon transgenic mice. Sci Rep.

[160] Trebak M., Kinet J.P. (2019). Calcium signalling in T cells. Nat Rev Immunol.

[161] Tomura M., Yoshida N., Tanaka J., Karasawa S., Miwa Y., Miyawaki A. (2008). Monitoring cellular movement in vivo with photoconvertible fluorescence protein "Kaede" transgenic mice. Proc Natl Acad Sci USA.

[162] Bernard C.C.A., Carnegie P.R. (1975). Experimental autoimmune encephalomyelitis in mice: immunologic response to mouse spinal cord and myelin basic proteins. J Immunol.

[163] Richert J.R., Lehky T.J., Muehl L.A., Mingoli E.S., McFarlin D.E. (1985). Myelin basic protein-specific T cell lines and clones derived from SJL mice with experimental allergic encephalomyelitis. J Neuroimmunol.

[164] Fritz R.B., Chou C.H.J., McFarlin D.E. (1983). Relapsing experimental allergic encephalomyelitis induced by myelin basic protein. J Immunol.

[165] Zamvil S.S., Nelson P.A., Trotter J., Mitchell D.J., Knobler R.L., Fritz R.B. (1985). T-cell clones specific for myelin basic protein induce chronic relapsing paralysis and demyelination. Nature.

[166] Sobel R.A., Van der Veen R.C., Lees M.B. (1986). The immunopathology of chronic experimental allergic encephalomyelitis induced in rabbits with bovine proteolipid protein. J Immunol.

[167] Tuohy V.K., Sobel R.A., Lees M.B. (1988). Myelin proteolipid protein-induced experimental allergic encephalomyelitis: variation of disease expression in different strains of mice. J Immunol.

[168] McRae B.L., Kennedy M.K., Tan L.J., Dal Canto M.C., Picha K.S., Miller S.D. (1992). Induction of active and adoptive relapsing experimental autoimmune encephalomyelitis (EAE) using an encephalitogenic epitope of proteolipid protein. J Neuroimmunol.

[169] Tuohy V.K., Lu Z., Sobel R.A., Laursen R.A., Lees M. (1989). Identification of an encephalitogenic determinant of myelin proteolipid protein for SJL mice. J Immunol.

[170] Whitham R.H., Bourdette D.N., Hashim G.A., Herndon G.M., Ilg R.C., Vandenbark A.A. (1991). Lymphocytes from SJL/J mice immunized with spinal cord respond selectively to a peptide of proteolipid protein and transfer relapsing demyelinating experimental autoimmune encephalomyelitis. J Immunol.

[171] McRae B.L., Vanderlugt C.L., Dal Canto M.C., Miller S.D. (1995). Functional evidence for epitope spreading in the relapsing pathology of experimental autoimmune encephalomyelitis. J Exp Med.

[172] Pöllinger B., Krishnamoorthy G., Berer K., Lassmann H., Bösl M., Dunn R. (2009). Spontaneous relapsing-remitting EAE in the SJL/J mouse: MOG-reactive transgenic T cells recruit endogenous MOG-specific B cells. J Exp Med.

[173] Kerlero de Rosbo N., Mendel I., Ben-Nun A. (1995). Chronic relapsing experimental autoimmune encephalomyelitis with a delayed onset and an atypical course, induced on PL/J mice by myelin oligodendrocyte glycoprotein (MOG)-derived peptide: preliminary analysis of MOG T cell epitopes. Eur J Immunol.

[174] Bettadapura J., Menon K.K., Moritz S., Liu J., Bernard C.C. (1998). Expression, purification, and encephalitogenicity of recombinant human myelin oligodendrocyte glycoprotein. J Neurochem.

[175] Reddy J., Illés Z., Zhang X.M., Encinas J.A., Pyrdol J., Nicholson L. (2004). Myelin proteolipid protein-specific CD4+CD25+ regulatory cells mediate genetic resistance to experimental autoimmune encephalomyelitis. Proc Natl Acad Sci USA.

